# Supplementary material for: GWAS and ExWAS of blood mitochondrial DNA copy number identifies 71 loci and highlights a potential causal role in dementia
Source: eLife. 2022 Jan 13;11:e70382. doi: 10.7554/eLife.70382 (PMC8865845; doi:10.7554/eLife.70382)
Supplement: Transparent reporting form [file elife-70382-transrepform1.docx]

***eLife’s* transparent reporting form**

We encourage authors to provide detailed information *within their submission* to facilitate the interpretation and replication of experiments. Authors can upload supporting documentation to indicate the use of appropriate reporting guidelines for health-related research (see [EQUATOR Network](http://www.equator-network.org/%20)), life science research (see the [BioSharing Information Resource](https://biosharing.org/)), or the [ARRIVE guidelines](http://www.plosbiology.org/article/info:doi/10.1371/journal.pbio.1000412) for reporting work involving animal research. Where applicable, authors should refer to any relevant reporting standards documents in this form.

If you have any questions, please consult our Journal Policies and/or contact us: [editorial@elifesciences.org](mailto:editorial@elifesciences.org).

**Sample-size estimation**

- You should state whether an appropriate sample size was computed when the study was being designed
- You should state the statistical method of sample size computation and any required assumptions
- If no explicit power analysis was used, you should describe how you decided what sample (replicate) size (number) to use

Please outline where this information can be found within the submission (e.g., sections or figure legends), or explain why this information doesn’t apply to your submission:

Power calculations were not performed for the mtDNA-CN GWAS, since the UK Biobank was a preexisting study with a fixed sample size. Sample size considerations were made for (i) phenome-wide association testing for *SAMHD1* rare mutation carriers and (ii) MR analyses with mitochondrial disease phenotypes. Outcomes were excluded if there were not enough cases to be well-powered as described in the Materials and Methods on page 28, lines 37-39 and page 29, lines 7-9.

**Replicates**

- You should report how often each experiment was performed
- You should include a definition of biological versus technical replication
- The data obtained should be provided and sufficient information should be provided to indicate the number of independent biological and/or technical replicates
- If you encountered any outliers, you should describe how these were handled
- Criteria for exclusion/inclusion of data should be clearly stated
- High-throughput sequence data should be uploaded before submission, with a private link for reviewers provided (these are available from both GEO and ArrayExpress)

Please outline where this information can be found within the submission (e.g., sections or figure legends), or explain why this information doesn’t apply to your submission:

Inclusion and exclusion criteria for AutoMitoC development, GWAS, and ExWAS are described in the Materials and Methods section on pages 22, 24-25, and 27-28, respectively. Phenotype definitions for the PheWAS is described in the Materials and Methods (page 29). Phenotype codes for FinnGen phenotypes used in the MR analyses are provided in Figure 6 – source data 1. Handling of outliers for blood cell counts is described in the Materials and Methods (page 21) and for the Mendelian Randomization (page 30). Replication of the MR finding for dementia is described in the Materials and Methods (page 30).

**Statistical reporting**

- Statistical analysis methods should be described and justified
- Raw data should be presented in figures whenever informative to do so (typically when N per group is less than 10)
- For each experiment, you should identify the statistical tests used, exact values of N, definitions of center, methods of multiple test correction, and dispersion and precision measures (e.g., mean, median, SD, SEM, confidence intervals; and, for the major substantive results, a measure of effect size (e.g., Pearson's r, Cohen's d)
- Report exact p-values wherever possible alongside the summary statistics and 95% confidence intervals. These should be reported for all key questions and not only when the p-value is less than 0.05.

Please outline where this information can be found within the submission (e.g., sections or figure legends), or explain why this information doesn’t apply to your submission:

Statistical analysis descriptions for the GWAS, ExWAS, PheWAS, and MR analyses are provided in Materials and Methods (pages 24-30). GWAS, ExWAS, PheWAS, and MR results are presented visually in Figures 3, 4A, 4B, and 6, respectively. Exact P-values and summary statistics are reported for the corresponding results in Supplementary File 2 Tables 1 & 2, Figure 4 – source data 1, Figure 4 – source data 2, and Figure 6 – source data 1, respectively.

(For large datasets, or papers with a very large number of statistical tests, you may upload a single table file with tests, Ns, etc., with reference to sections in the manuscript.)

**Group allocation**

- Indicate how samples were allocated into experimental groups (in the case of clinical studies, please specify allocation to treatment method); if randomization was used, please also state if restricted randomization was applied
- Indicate if masking was used during group allocation, data collection and/or data analysis

Please outline where this information can be found within the submission (e.g., sections or figure legends), or explain why this information doesn’t apply to your submission:

Not applicable. In this study, investigators did not perform group allocation.

**Additional data files (“source data”)**

- We encourage you to upload relevant additional data files, such as numerical data that are represented as a graph in a figure, or as a summary table
- Where provided, these should be in the most useful format, and they can be uploaded as “Source data” files linked to a main figure or table
- Include model definition files including the full list of parameters used
- Include code used for data analysis (e.g., R, MatLab)
- Avoid stating that data files are “available upon request”

Please indicate the figures or tables for which source data files have been provided:

Genome-wide significant SNPs (Supplementary File 2 – Tables 1-2), ExWAS results (Figure 4 – source data 1), PheWAS results (Figure 4 – source data 2), genetic instruments for MR analyses (Supplementary File 2 – Tables 1 and 8), and MR results (Figure 6 – source data 1) have been provided.

Individual-level data cannot be provided, but it is available to all researchers by application to the UK Biobank. Individual-level AutoMitoC estimates for UK Biobank have been returned to the UK Biobank Access Team for use by other approved researchers.

Code for AutoMitoC is available here: <https://github.com/GMELab/AutoMitoC> including a subsample of the validation dataset with individual-level data (age, sex, qPCR mtDNA-CN). Full mtDNA-CN genome-wide summary statistics have been uploaded to the GWAS catalogue:

*GCST90026371 (Trans-ethnic meta-analysis:* [*http://ftp.ebi.ac.uk/pub/databases/gwas/summary_statistics/GCST90026001-GCST90027000/GCST90026371/*](http://ftp.ebi.ac.uk/pub/databases/gwas/summary_statistics/GCST90026001-GCST90027000/GCST90026371/)*)*

*GCST90026372 (European:* [*http://ftp.ebi.ac.uk/pub/databases/gwas/summary_statistics/GCST90026001-GCST90027000/GCST90026372/*](http://ftp.ebi.ac.uk/pub/databases/gwas/summary_statistics/GCST90026001-GCST90027000/GCST90026372/)*)*

*GCST90026373 (South Asian:* [*http://ftp.ebi.ac.uk/pub/databases/gwas/summary_statistics/GCST90026001-GCST90027000/GCST90026373/*](http://ftp.ebi.ac.uk/pub/databases/gwas/summary_statistics/GCST90026001-GCST90027000/GCST90026373/)*)*

*GCST90026374 (African:* [*http://ftp.ebi.ac.uk/pub/databases/gwas/summary_statistics/GCST90026001-GCST90027000/GCST90026374/*](http://ftp.ebi.ac.uk/pub/databases/gwas/summary_statistics/GCST90026001-GCST90027000/GCST90026374/)*).*
